# Noninvasive, longitudinal imaging-based analysis of body adipose tissue and water composition in a melanoma mouse model and in immune checkpoint inhibitor-treated metastatic melanoma patients

**DOI:** 10.1007/s00262-020-02765-8

**Published:** 2020-11-01

**Authors:** Wolfgang M. Thaiss, Sergios Gatidis, Tina Sartorius, Jürgen Machann, Andreas Peter, Thomas K. Eigentler, Konstantin Nikolaou, Bernd J. Pichler, Manfred Kneilling

**Affiliations:** 1 Department of Preclinical Imaging and Radiopharmacy, Werner Siemens Imaging Center, Eberhard Karls University, 72076 Tübingen, Germany; 2 Department of Diagnostic and Interventional Radiology, Eberhard Karls University, 72076 Tübingen, Germany; 3 iFIT-Cluster of Excellence, Eberhard Karls University, 72076 Tübingen, Germany; 4 German Center for Diabetes Research (DZD E.V.), Neuherberg, Germany; 5 Institute for Diabetes Research and Metabolic Diseases of the Helmholtz Centre Munich at the University of Tübingen, Tübingen, Germany; 6 Section of Experimental Radiology, Department of Diagnostic and Interventional Radiology, University Hospital Tübingen, Tübingen, Germany; 7 Department for Diagnostic Laboratory Medicine, Institute for Clinical Chemistry and Pathobiochemistry, University Hospital Tübingen, Tübingen, Germany; 8 Department of Dermatology, University Hospital Tübingen, Liebermeisterstreet 20, 72076 Tübingen, Germany; 9 German Cancer Consortium (DKTK), German Cancer Research Center (DKFZ) Partner Site Tübingen, 72076 Tübingen, Germany; 10 Present Address: Department of Nuclear Medicine, University of Ulm, Albert-Einstein-Allee 23, 89081 Ulm, Germany

**Keywords:** Melanoma, Cancer cachexia, MRI, Segmentation, Therapy monitoring, Immune checkpoint inhibitor therapy

## Abstract

**Background:**

As cancer cachexia (CC) is associated with cancer progression, early identification would be beneficial. The aim of this study was to establish a workflow for automated MRI-based segmentation of visceral (VAT) and subcutaneous adipose tissue (SCAT) and lean tissue water (LTW) in a B16 melanoma animal model, monitor diseases progression and transfer the protocol to human melanoma patients for therapy assessment.

**Methods:**

For in vivo monitoring of CC B16 melanoma-bearing and healthy mice underwent longitudinal three-point DIXON MRI (days 3, 12, 17 after subcutaneous tumor inoculation). In a prospective clinical study, 18 metastatic melanoma patients underwent MRI before, 2 and 12 weeks after onset of checkpoint inhibitor therapy (CIT; *n* = 16). We employed an in-house MATLAB script for automated whole-body segmentation for detection of VAT, SCAT and LTW.

**Results:**

B16 mice exhibited a CC phenotype and developed a reduced VAT volume compared to baseline (B16 − 249.8 µl, − 25%; controls + 85.3 µl, + 10%, *p* = 0.003) and to healthy controls. LTW was increased in controls compared to melanoma mice. Five melanoma patients responded to CIT, 7 progressed, and 6 displayed a mixed response. Responding patients exhibited a very limited variability in VAT and SCAT in contrast to others. Interestingly, the LTW was decreased in CIT responding patients (− 3.02% ± 2.67%; *p* = 0.0034) but increased in patients with progressive disease (+ 1.97% ± 2.19%) and mixed response (+ 4.59% ± 3.71%).

**Conclusion:**

MRI-based segmentation of fat and water contents adds essential additional information for monitoring the development of CC in mice and metastatic melanoma patients during CIT or other treatment approaches.

## Introduction

Cancer cachexia (CC) as an epiphenomenon associated with cancer and other chronic diseases is defined by an involuntary loss of muscle and fat mass [1]. It can be associated with a variety of diseases, such as chronic heart failure, chronic kidney disease, and cancer. In the latter case, CC is one of the major causes of death of tumor patients.

While several factors have been proposed to influence the development of CC, the main drivers leading to this phenomenon are still poorly understood. In fact, even within the same cancer entity, a broad variation in the presence or absence of CC is reported. Among the main drivers, brown adipose tissue (BAT) [2] and inflammatory factors, such as interleukins, have been discussed [3–6] and recent advances in research have identified novel protein functions [7] and some hormones, e.g., PTHrP [8], as potential factors.

Our understanding of the mechanisms of CC that lead to reduced quality of life and mechanisms of resilience in tumor patients and possible interventions is increasing [9–12]. Thus, the challenge for the treating oncologist is not only the cancer treatment as such, but also to identify patients at risk for developing CC, as the initial changes might be subclinical, especially in an increasingly obese society. A consensus definition has outlined three stages of CC, with a refractory cachectic stage in which failure of all interventional attempts leads to further weight loss and morbidity, independent of the accompanying cancer treatment [1, 4].

Imaging modalities, such as computed tomography (CT) and magnetic resonance imaging (MRI), are routinely used for cancer staging in oncology. Such examinations mainly focus on the detection of the primary tumor and metastases and their changes in response to therapy. However, recently, additional quantitative methods have been developed that use more of the information at hand, such as the segmentation and mass of muscle and adipose tissue [13–17]. As this information can be extracted from whole-body staging examinations, automated analysis would provide valuable information for the treating physician.

Recent large-scale studies of breast cancer patients found prognostic value in the presence of clinically non-evident muscle wasting detected with routine CT scans for staging, even in nonmetastatic patients and in a setting of changes of subcutaneous adipose tissue [18, 19]. Other studies have investigated the prognostic value of body composition in association with metabolic risk factor profiles or adipokine levels, and found it added value to clinical decision making and showed a prognostic impact [20–22]. Thus, imaging appears to be able to contribute predictive information that might not otherwise be accessible. However, none of these studies investigated the possible contribution of longitudinal monitoring of changes of body composition under checkpoint inhibitor therapy (CIT).

To better understand the development of CC, several mouse models have been described over the last few decades to study the phenomenon observed in humans. While spontaneously developing cancer models most closely resemble human carcinogenesis and its associated changes in metabolism and immune function, they are difficult to monitor noninvasively in terms of their cachectic stage. While inferior in terms of their comparability with human physiology, subcutaneous exogenous tumor models have the advantages of comparable tumor growth rates and weight loss within a cohort. The B16 melanoma model has been extensively studied in the past few decades and is a widely used experimental model to study CC [23, 24]. Exogenous B16 melanomas will grow in immunocompetent C57BL/6 J mice, which seems to be an essential factor with regard to the suggested role of immunological factors in CC development.

In a recent study, we noninvasively investigated changes in glucose metabolism in vivo (employing multimodal ^18^F-FDG-PET/CT or PET/MRI) in primary and secondary lymphatic organs of immune checkpoint inhibitor-treated experimental mice as well as metastatic melanoma patients and identified differential signatures enabling us to differentiate between responders and nonresponders [25].

In this study, we aimed to establish a workflow for automated MRI-based segmentation of subcutaneous, visceral adipose tissue and lean body mass that is applicable for both preclinical CC studies in rodents and in metastatic cancer patients. This should allow for early identification of CC and monitoring of the progression of CC as well as monitoring body adipose tissue and water composition changes in cases of successful treatment or treatment failure for therapeutic regimes, such as CIT.

## Methods

All animal experiments were performed according to the German Animal Protection Law with permission from the local authorities (Regierungspräsidium Tübingen, Germany). The prospective human study was approved by the appropriate local ethics committee and has been performed in accordance with the ethical standards laid down in the 1964 Declaration of Helsinki and its later amendments.

### Preclinical study

#### Animals

In this study, we used 8- to 10-week-old female C57BL/6 J mice (Charles River Laboratories, Sulzfeld, Germany). The mice were kept under standardized conditions in isolated ventilated cages (20 ± 1 °C room temperature, 50 ± 10% relative humidity, and a 12 h light–dark cycle) with free access to a standard diet for rodents (fed ad libitum) and tap water.

#### Cell culture

B16-F10 melanoma tumor cells (Perkin Elmer, Waltham, United States) were cultured in RPMI 1640, with 100 U/mL penicillin, 100 mg/L streptomycin and 10% fetal calf serum (all from Biochrom GmbH, Berlin, Germany) at 37 °C in a humidified atmosphere of 5% CO_2_ in a cell culture cabinet (HeraSafe KS18, Thermo/Kendro, Dreieich/Hanau, Germany).

#### Experimental B16 melanoma model

To induce CC in the mice, 0.5 × 10^6^ B16 melanoma tumor cells (Perkin Elmer, Waltham, United States) were injected subcutaneously into the lateral abdomen and the progression of CC was monitored by weight loss. The animals were weighed daily to monitor disease progression. For weight comparison to the control group, the tumor weight was identified after the last measurement by removing it and weighing it, and subtracting its weight from the final whole body weight.

#### In vivo MR studies

C57BL/6 J mice underwent MR measurements in a 7 T small animal MRI scanner (Clinscan, Bruker Biospin, Ettlingen, Germany) 3, 12 and 17 days after tumor inoculation. Animals were anesthetized with 1.5% isoflurane (CP-Pharma, Burgdorf, Germany) evaporated in oxygen at a flow rate of 0.5 L/min. Body temperature was measured continuously and regulated by a warming pad, and anesthesia depths were monitored by breathing frequency. The following scan parameters were used: whole-body imaging was performed using a 2D gradient echo sequence with 3 echoes (1.26, 1.6, 1.94 ms) and the following parameters: slice thickness 2 mm, matrix 162 × 192; FOV 38 × 45; repetition time 450 ms, flip angle 20°. Decomposition of fat and water images from the acquired MR data was performed using in-house software (implemented in MATLAB, version 2014b) based on a method previously described by Berglund et al. [26]. Subsequently, separation of VAT, SCAT and LTW was performed using in-house software based on active contour segmentation (implemented in MATLAB, version 2014b) as previously described in [27] and outlined below. For the analysis, the subcutaneous tumor was excluded.

#### Blood samples

After the last MRI scan, 17 days after tumor inoculation, the mice were sacrificed and blood samples were prepared, centrifuged and frozen at – 80 °C before analysis of cholesterol, low-density lipoprotein cholesterol (LDL-C), triacylglycerols (TAG) and total protein was carried out using an automated clinical chemistry analyzer ADVIA 1800 (Siemens Healthcare Diagnostics, Eschborn, Germany).

### Prospective clinical study

#### Patient population

All participants gave written informed consent prior to entry into the study. Between 09/2014 and 10/2016, 18 patients with Stage IV melanoma were prospectively included before initiation of systemic immune therapy and their data were retrospectively analyzed. All patients were examined at 3 time points: before treatment, and at 2 and 12 weeks after initiation of treatment. The patients were categorized by therapeutic response as responder, mixed responder or nonresponder in accordance with the clinical response evaluation.

#### MR study

All whole-body MR examinations were performed as part of a clinically indicated FDG-PET/MR staging examination on a combined 3 T PET/MR scanner (Siemens Biograph mMR, Siemens Healthcare, Erlangen, Germany). The study MR protocol consisted of a whole-body dual echo Dixon MR sequence in the axial orientation (skull base to mid-thigh level) with the following parameters: resolution 2.6 × 2.6 mm, slice thickness 3.1 mm, echo times 1.32, 2.46 ms, repetition time 3.96 ms, flip angle 9°. The data were processed as described above.

#### MR data analysis

In animal studies as well as patient examinations, adipose tissue compartments (subcutaneous (SCAT) and visceral adipose tissue (VAT)) as well as the lean tissue water compartment (LTW)) were automatically segmented on the fat images using an active contour-based approach as previously described by Wuerslin et al. [27]. In short, this method is based on the use of active contours (so-called snake algorithm) to detect the boundary between SCAT and the underlying musculature/fascia. The outer contour of the skin is thereby used as initial contour for the subsequent iterative inward-pointing propagation of the contour. The energy of the contour finds a local minimum at the border between SCAT and muscle tissue/fascia on fat images due to a sharp signal edge along this border. The algorithm was applied to each single 2D slice separately from the skull base to the mid-thigh level. Automated segmentation results were validated visually, and corrections of segmentations were performed where necessary. No specific adaptation of the algorithm was necessary for translation from human to animal data as the matrix size (and thus the step size of the snake algorithm) was comparable between human and animal data and as the fat/water contrast was also similar between human and animal data. This was determined empirically.

### Statistics

Values are given as mean ± standard deviation. Changes in body weight, fat and water content are given in percentages. GraphPad Prism 7.03, GraphPad Software, La Jolla California USA was used for the statistical analysis. A normal distribution was confirmed using the Shapiro–Wilk normality test. For imaging data, measurements in follow-up examinations were expressed as percent change from baseline to correct for individual baseline values and used for statistical analysis. One-way ANOVA was used for group comparisons and *p* values were corrected for multiple testing with Bonferroni measures. Pearson’s *r* was used for correlation analysis. Unpaired Student’s *t* test was used for two-group comparisons and for parameters from the blood sampling, and values < 0.05 were considered significant. Graphs were produced with MATLAB 9.5 R2018b (The MathWorks Inc., Natick, Massachusetts) and GraphPad Prism 7.03.

## Results

### Determination of body composition and cachexia monitoring in the experimental B16 melanoma mouse model

**Fig. 1:**
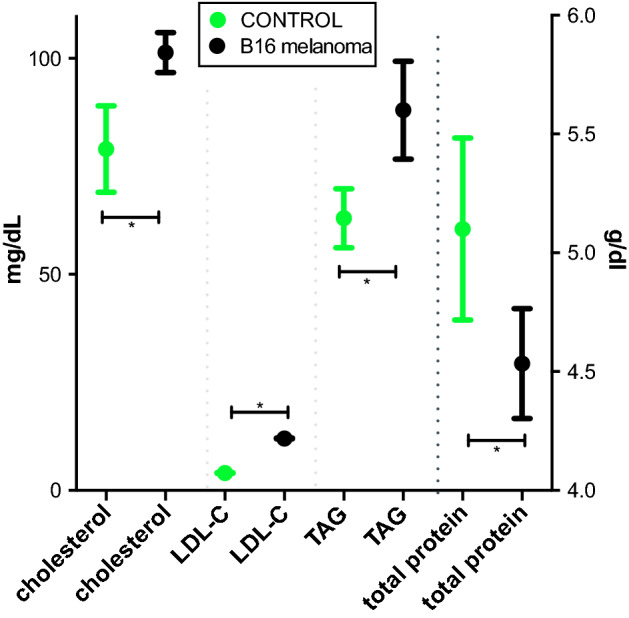
Serum parameters 17 days after tumor inoculation. Total cholesterol, LDL-C (low-density lipoprotein cholesterol), TAG (triacylglycerols) and total protein 17 days after subcutaneous B16 melanoma cell inoculation (*n* = 7) compared to healthy control animals (*n* = 6). Values are given in mg/dl and g/dl, respectively, with mean and standard deviation. Significant group differences are present for all parameters (*p* < 0.0001, unpaired Student’s *t*-test)

A cachexia phenotype was confirmed by monitoring animal weight and characteristic changes in serum parameters. Seventeen days after tumor inoculation, the weight of the B16 melanoma-bearing animals (*n* = 7) was 18.7 ± 1.3 g compared to 20.3 ± 0.4 g in the healthy control group (*n* = 6, *p* = 0.04). Blood samples acquired at day 17 demonstrated a cachectic phenotype in the tumor-bearing animals with cholesterol levels of 79 ± 10 mg/dl compared to 101 ± 5 mg/dl in the control animals (*p* < 0.02). LDL-C in the cachexia mice (12 mg/dl) was increased threefold when compared to healthy control mice (4 mg/dl, *p* < 0.0001; Fig. 1a). In line with these data, we also found a significant increase in TAG in the tumor-bearing mice (88 ± 8 mg/dl) in comparison to the control mice (63.0 ± 3.4 mg/dl, *p* = 0.02, Fig. 1).

**Fig. 2:**
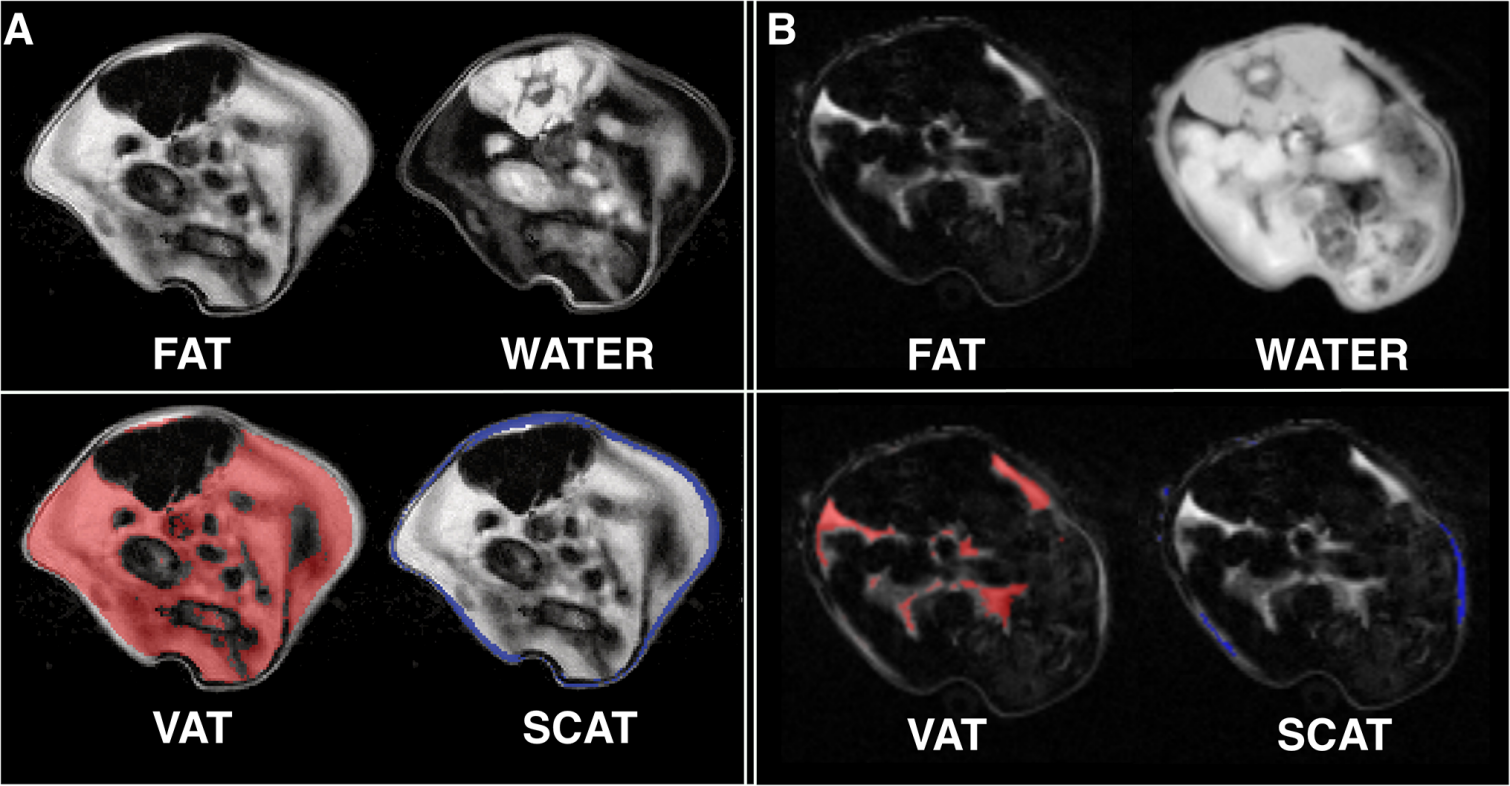
MRI-based segmentation in the B16 melanoma mouse model. Representative example for Dixon-based whole-body segmentation in a 10-week-old mouse at baseline (**a**) and 17 days after B16 melanoma inoculation with a cachectic phenotype (**b**), axial slices of the lumbar region. Fat and water MRI images (top row) as well as delineated segments of visceral adipose tissue (VAT, bottom left with VAT indicated in red) and subcutaneous adipose tissue (SCAT, bottom right with SCAT indicated in blue) are shown

**Fig. 3:**
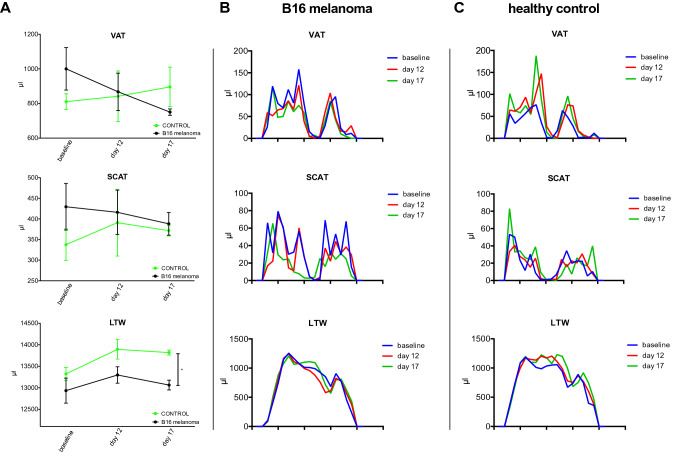
MRI-based segmentation results in melanoma mice and healthy control mice. **a** Quantitative MRI-measurements of visceral adipose tissue (VAT, top) and subcutaneous adipose tissue (SCAT, middle) as well as lean tissue water (LTW, bottom) distribution in mice with B16 melanoma (*n* = 7) and healthy control animals (*n* = 6) at baseline, after 12 and 17 days outlined in the caudocranial distribution. Melanoma mice showed reduced VAT volume compared to baseline (B16 − 249.8 µl, − 25%; *p* = 0.003) and to healthy controls (+ 85.3 µl, + 10%; *p* = 0.26 for group comparisons after 17 days, n.s.). LTW was slightly increased in controls compared to melanoma-bearing mice (13,817.5 ± 115.7 µl and 13,063.0 ± 227.2 µl, *p* = 0.049). B, C give examples of the MRI segmentation with delineation of VAT, SCAT and LTW in a B16 melanoma-bearing mouse (**b**) and a healthy control mouse (**c**) at baseline and after 12 and 17 days

We conducted automated segmentation of VAT and SCAT, as well as LTW, of all experimental B16 melanoma-bearing and healthy control animals. An example of the segmentation is given in Fig. 2. B16 melanoma-bearing animals exhibited a volume of VAT at baseline (3 days after tumor inoculation) of 1000.1 ± 323.0 µl, while VAT in the control animals was 810.7 ± 111.6 µl (n.s.; Fig. 3a). After 12 days, VAT in the melanoma animals was reduced to 867.7 ± 285.1 µl (− 12% from baseline), while healthy controls showed a VAT volume of 841.9 ± 357.8 µl (+ 4% from baseline). After 17 days, VAT was reduced in the melanoma group by a mean of 249.8 µl (− 25% from baseline, *n* = 7; *p* = 0.003 to baseline), while in the healthy control animals, VAT increased with age (85.3 µl; + 10% from baseline, *n* = 6; *p* = 0.26 for the group comparison after 17 days, n.s.; Fig. 3a).

SCAT volume in the B16-bearing and healthy control animals was 429.6 ± 149.3 µl and 337.6 ± 93.3 µl, respectively, at baseline (n.s.). After 12 days, the SCAT volume in the melanoma mice was 416.1 ± 142.4 µl (− 2% from baseline) and 390.9 ± 197.7 µl in the healthy control mice (+ 15% from baseline, n.s.). After 17 days, the SCAT volume in the B16 melanoma mice was reduced to 388.2 ± 55.0 µl (− 16% from baseline) and to 372.7 ± 25.7 µl in the control animals (+ 10% from baseline, n.s.).

In addition, the lean tissue water volume changed from 12,934.5 ± 768.3 µl (at baseline) to 13,297.6 ± 503.9 µl (day 12) and to 13,063.0 ± 227.2 µl (day 17) in the B16-bearing animals and from 13,322.8 ± 369.1 µl to 13,893.3 ± 553.6 µl (day 12) and to 13,817.5 ± 115.7 µl (day 17) in the control animals (*p* = 0.049). Figure 3b, c gives examples of the MRI segmentation with delineation of VAT, SCAT and LTW in a B16 melanoma-bearing mouse (B) and a healthy control mouse (C).

### Identification and monitoring of CC in immune checkpoint inhibitor-treated patients with metastatic melanoma

In a prospective clinical study, eighteen patients (10 male and 8 female patients with a mean age of 62 ± 10 years) underwent MR scans at baseline (before therapy) and follow-up MR investigations after two weeks and 12 weeks after onset of PD-1- or CTLA-4 immune checkpoint inhibitor treatment (*n* = 16) or targeted therapy with BRAF inhibitors (*n* = 2). In addition, we determined the patient weight and body mass index at baseline and in both follow-up examinations (Tables 1, 2). Automated segmentation of VAT, SCAT and LWT was feasible in all cases except for one patient due to a metallic hip prosthesis. In this case, the affected region was excluded for all three examinations.

**Table 1:** Summary of melanoma patient data for body weight (BW) and body mass index (BMI) at baseline and after 2 weeks and 12 weeks of treatment

Patient	BW at baseline [kg]	BW after 2 weeks [kg]	BW after 12 weeks [kg]	BMI at baseline [kg/m^2^]	BMI after 2 weeks [kg/m^2^]	BMI after 3 months [kg/m^2^]	VAT change from baseline (%)	SCAT change from baseline (%)	LTW change from baseline (%)	Therapy	Response
1	90.9	85.0	79.9	26.0	24.3	22.8	− 44.83	− 34.87	− 1.31	PD-1-mAb	PD
2	96.2	100.3	100.8	32.5	33.9	34.1	4.86	0.26	1.11	CTLA-4-mAb	PD
3	85.7	85.9	89.3	31.5	31.6	32.8	0.32	4.04	8.59	PD-1-mAb	MIXED
4	57.2	57.5	54.7	22.3	22.5	21.4	− 5.76	− 3.78	1.65	PD-1-mAb	PD
5	82.6	82.0	80.6	27.9	27.7	27.2	− 14.50	− 7.48	1.76	PD-1-mAb	MIXED
6	84.5	85.0	86.7	26.1	26.2	26.8	− 4.07	− 2.48	− 5.72	CTLA-4-mAb	RTT
7	67.4	66.0	66.4	24.8	24.2	24.4	1.96	− 0.49	− 4.13	PD-1-mAb	RTT
8	85.1	84.0	79.0	27.5	27.1	25.5	− 38.13	− 17.07	6.76	PD-1-mAb	MIXED
9	100.3	99.0	96.2	32.8	32.3	31.4	− 6.44	− 4.58	− 0.31	PD-1-mAb	MIXED
10	93.1	92.1	92.9	28.1	27.8	28.0	1.88	− 0.72	1.42	PD-1-mAb	RTT
11	71.3	72.0	70.0	25.0	25.2	24.5	12.96	− 0.06	2.31	BRAF	PD
12	61.2	60.0	62.3	22.5	22.0	22.9	− 3.50	2.69	4.80	PD-1-mAb	PD
13	65.7	68.3	67.3	21.2	22.0	21.7	16.67	13.85	1.54	PD-1-mAb	PD
14	85.3	86.2	86.5	24.4	24.7	24.7	1.96	1.25	3.04	PD-1-mAb	RTT
15	87.6	82.0	75.5	28.6	26.8	24.7	− 13.86	− 19.49	− 14.67	BRAF	MIXED
16	112.5	110.0	113.0	32.5	31.8	32.7	− 1.16	− 8.36	4.00	PD-1-mAb	PD
17	82.2	83.0	83.3	25.4	25.6	25.7	7.17	4.89	− 3.62	PD-1-mAb	RTT
18	53.6	55.7	55.1	20.0	20.7	20.5	17.13	7.42	6.15	PD-1-mAb	MIXED

The percent changes in visceral adipose tissue (VAT), subcutaneous adipose tissue (SCAT) and lean tissue water (LTW) from baseline to the last measurement are given. The response assessment is given for the progressive disease (PD), mixed response to treatment (MIXED) and response to treatment (RTT) groups

**Table 2:**
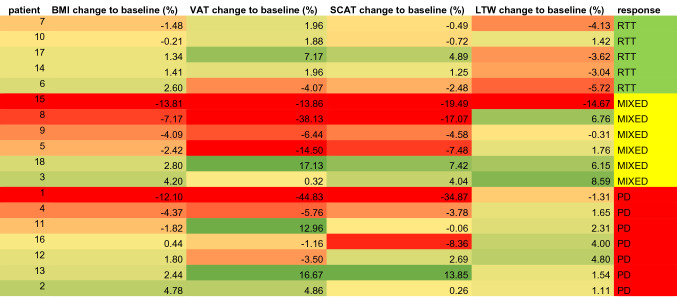
Changes for BMI, VAT, SCAT and LTW for all patients categorized by response assessment for progressive disease (PD), mixed response to treatment (MIXED) and response to treatment (RTT); green: increase; red: decrease, yellow: no change

Five patients were classified as immunotherapy responders after 12 weeks (RTT), six patients demonstrated a mixed response (MIXED), whereas seven patients showed progressive disease (PD, Table 1). BMI changed with a mean of + 0.73% ± 1.59% for patients with RTT, − 4.66% ± 6.89% for MIXED and + 0.55% ± 3.25% for patients with PD.

The change in body weight in percent from baseline to the examination after 12 weeks of therapy correlated well with the change in total fat (*r* = 0.81, *p* < 0.0001). No correlation was observed between the response to therapy and changes in BMI.

There were individual and overall differences in the composition and body fat changes, but no correlation was observed between changes in body weight and therapy response (responder: change from baseline mean 0.7% ± 1.6%, range − 1.5% and + 2.6%, nonresponder: − 1.3% ± 5.1%, range − 12.1% to + 4.8%).

LTW was increased in patients with a mixed response (+ 4.59% ± 3.71%) and progressive disease (+ 1.97% ± 2.19%) under CIT after 3 months compared to RTT (− 3.02% ± 2.67%, *n* = 16, *F* = 9.05, *p* = 0.0034; mean group difference for RTT vs. MIXED − 7.61 [95% CI − 12.4 to − 2.8] and for RTT vs. PD − 4.98 [95% CI − 9.6 to − 0.4], MIXED vs. PD n.s.).

Determination of VAT and SCAT revealed no significant differences among responders, mixed responders and nonresponders. However, while patients with a response to treatment showed a relatively narrow band of changes in VAT and SCAT after 12 weeks, the standard deviation and range of extreme changes increased with a less favorable response to treatment for both VAT and SCAT (Fig. 5c).

**Fig. 4:**
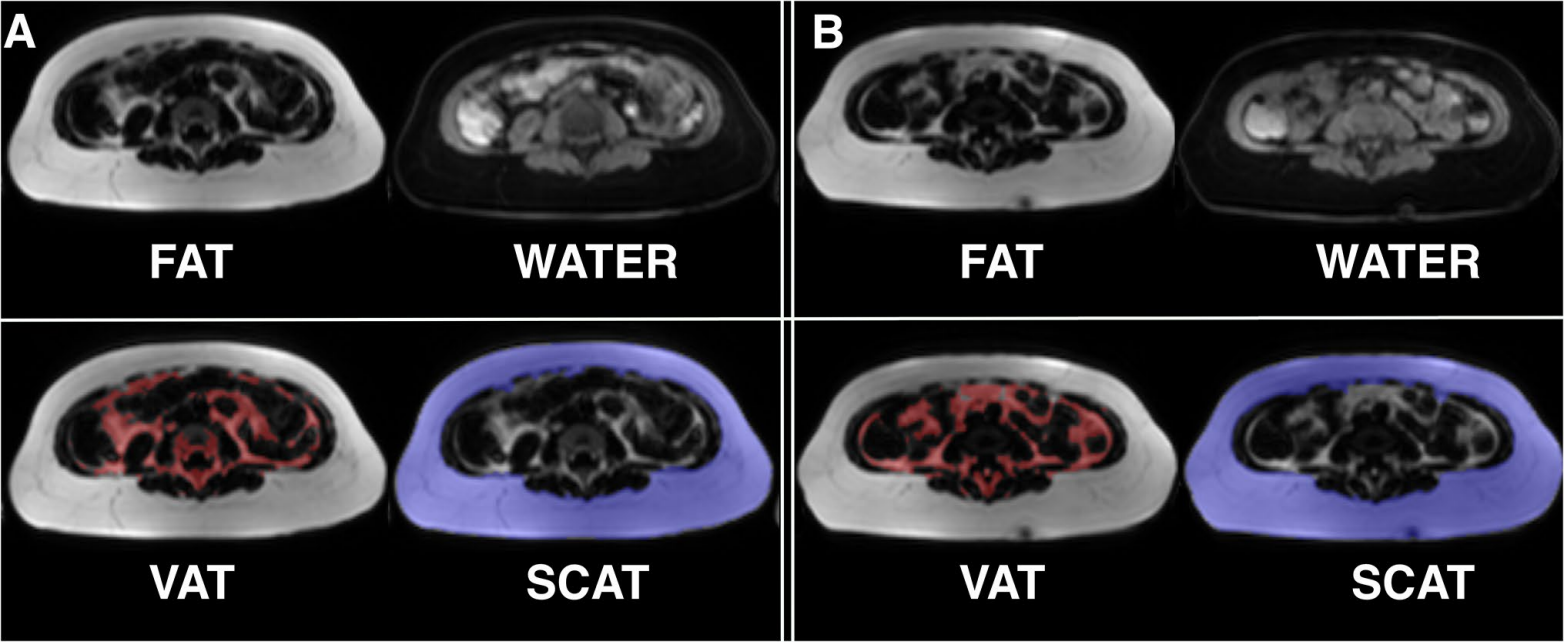
MRI-based segmentation in human melanoma patients treated with an immune checkpoint inhibitor. Representative example for Dixon-based whole-body segmentation of a 64-year-old melanoma patient with PD-1-mAbs therapy at baseline (**a**) and with progressive disease after 3 months of CIT (**b**), axial slices of the lumbar region are given. Fat and water MRI images (top row) as well as delineated segments of visceral adipose tissue (VAT, bottom left with VAT indicated in red) and subcutaneous adipose tissue (SCAT, bottom right with SCAT indicated in blue) are shown. Total fat was reduced by − 26% after 12 weeks, body weight was reduced by − 7.2%

**Fig. 5:**
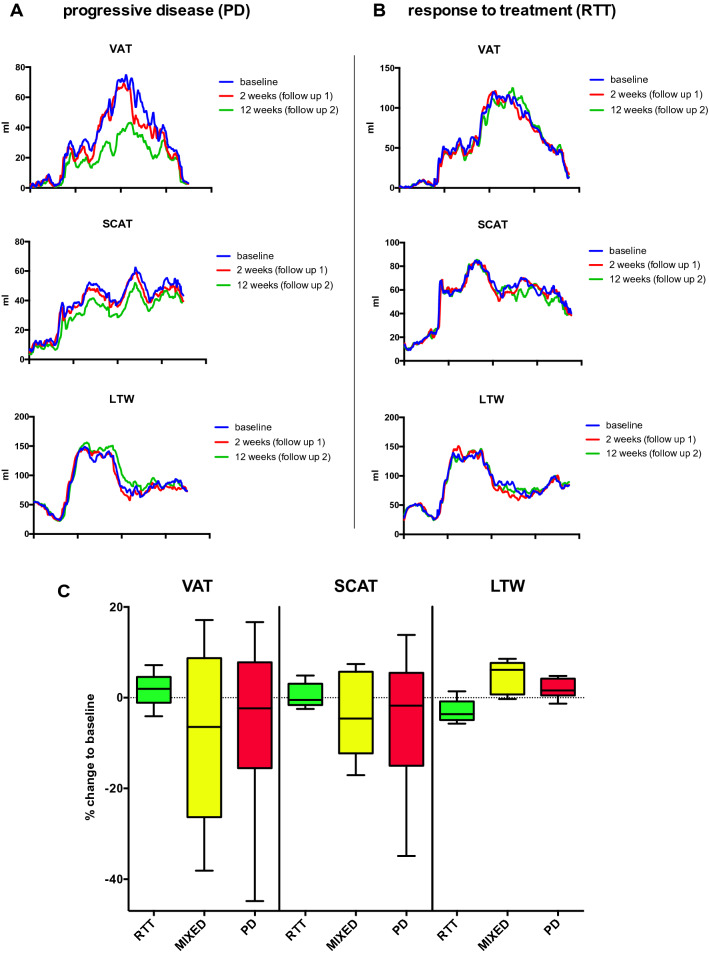
Representative examples of MRI-based whole-body segmentation and treatment-associated changes in metastatic melanoma patients treated with an immune checkpoint inhibitor. **a**, **b** Quantitative MRI measurements of visceral adipose tissue (VAT, top), subcutaneous adipose tissue (SCAT, middle) and lean tissue water (LTW, bottom) distribution in melanoma patients treated with CIT (*n* = 16). Examples for a melanoma patient with progressive disease (PD, **a**, same patient as in Fig. 4) and response to treatment (RTT, B) are given. Graphs show measurements at baseline and 2 weeks and 12 weeks into treatment outlined as the craniocaudal distribution. **c** Bar charts with percent change in body composition after 12 weeks of CIT therapy relative to baseline. The percent change in visceral adipose tissue (VAT), subcutaneous adipose tissue (SCAT) and lean tissue water (LTW) from baseline to the last measurement, stratified by therapeutic response, are given. No significant changes were present between groups for VAT and SCAT. Significant group differences were present for LTW (*F* = 9.05, *p* = 0.0034)

Figures 4 and 5a give a representative example of the MRI segmentation in a 64-year-old patient with rapidly progressive disease under CIT (nivolumab). The body weight of the patient was 85.1 kg at baseline. The follow-up examinations revealed a significant loss of the initial amount of total fat from 27,780 ml to 26,103 ml after 2 weeks (− 6%) and to 20,530 ml after 3 months (− 26%, Fig. 5a). In line with these data, body weight was reduced after two weeks to 84.0 kg (− 1.3%) and after 12 weeks to 79.0 kg (− 7.2% from baseline).

Conversely, Fig. 5b shows a representative patient with a response to treatment (complete remission) under CIT (nivolumab) with unaffected weight. The initial body weight was 93.1 kg and the total body fat volume was 38,347 ml. After two weeks, the patient exhibited an almost unaffected body weight of 92.1 kg (− 1.1%) and after 12 weeks of 92.9 kg (− 0.2% in total). Total body fat after two weeks of immunotherapy was 39,544 ml (+ 3.1%) and 38,664 ml after 12 weeks (+ 0.8% total).

## Discussion

In this study, we used an automated workflow for MRI-based quantification of visceral and subcutaneous adipose tissue and lean tissue water in mice and humans to noninvasively monitor changes in the development of CC in melanoma-bearing mice and to investigate changes in body composition in patients with metastasized melanoma under immune therapy. In both animals and metastatic melanoma patients, the quantification of visceral and subcutaneous fat was feasible.

The cachectic phenotype was confirmed in the mice by weight loss and serum parameters. While the effects of tumor induced cachexia on plasma lipids vary between different cancer types, both human and murine tumor cell lines lead to hyperlipidemia and can contribute to tumor growth [28].

The field of noninvasive fat-muscle quantification is of increasing interest. Starting from phantom studies [29], moving to feasibility studies in humans and animals [30, 31] and then to large-scale studies [32–34], multi-echo DIXON approaches have shown to be a robust and reliable method for fat and water quantification [35]. While DIXON techniques are part of many clinical examinations of the abdomen, segmentation of fat and body water is not performed routinely.

With the help of an automated segmentation script, body composition analysis was feasible for both human and murine subjects at different field strengths and with different scanner types. The equality of different fat segmentation strategies has recently been revisited [31, 36], making different approaches usable in broader clinical practice. Multi-point Dixon sequences have gained widespread use in clinical practice over the last years. While they show several advantages, such as the ability to adaptable echo times, more robust correction for magnetic field inhomogeneities or benefits in obese patients [35, 37, 38], we used the 2-point Dixon as this sequence is routinely implemented as part of a PET/MR protocol for the purpose of MR-based attenuation correction. The sequence is optimized for the scanner used in this study and robust against fat/water swaps. Furthermore, this sequence can be acquired in breath hold which is important for thoracic and abdominal acquisition. To reduce scan time, and as the 2-point Dixon segmentation was robust for the purpose at hand, we did not acquire an additional 3-point Dixon sequence. Most clinically oriented studies at hand focus on obesity-associated subjects [39], with predictive impacts for patient care. Rospleszcz et al. emphasized the need for longitudinal assessment in cardiometabolic risk estimation [22]. For tumor patients, recent studies with large cohorts of nonmetastatic breast cancer patients by Bradshaw and Caan [18, 19] demonstrated these image analysis methods added additional prognostic information in the detection of sarcopenia and adipose tissue distribution as these are not easily assessable clinically.

With the introduction of more immune checkpoint inhibitor-based therapeutic regimens in clinical practice, the need for early therapeutic response assessment by imaging is of increasing importance. A series of studies with a focus on imaging in the context of CIT investigated side effects and induced autoimmune phenomena, such as pancreatitis and myositis [40, 41]. Recently, monitoring of systemic immune responses and prediction of long-term outcomes were demonstrated for 18F-FDG-PET/CT [42] in ipilimumab- and nivolumab-treated melanoma patients. Despite the ease of direct assessment of therapeutic success of viable tumor tissue or the detection of side effects, the analysis of concomitant effects in immune therapy remains elusive.

Indirect patterns of favorable or unfavorable tumor therapy-associated metabolic signs of treatment success or tumor therapy-associated adverse metabolic consequences are becoming even more relevant with the use of CTI. CC, as an independent factor impacting cancer patient survival, is a tumor- and therapy-associated risk that is not easily assessable with clinical examinations and blood parameters [4].

We have recently investigated metabolic epiphenomena in melanoma patients receiving CIT and found increased metabolic activity in the bone marrow of patients with a response to CTI compared to that in nonresponders [25]. Seeking additional auxiliary metabolic indications for therapeutic efficacy, the aim of this study was to investigate changes in VAT and SCAT as a profile of metabolic therapy response. Thus, we adjusted automated segmentation from whole-body MRI-data to create a robust workflow for human and murine use and to monitor melanoma-associated metabolic phenomena, namely cachexia development and metabolic changes during CIT therapy in advanced melanoma.

We were able to observe the progress of CC in B16 melanoma-bearing animals and demonstrate a mean reduction of 25% of total visceral adipose tissue volume after 17 days. Monitoring the changes in metastatic melanoma patients revealed limited changes with regard to BMI; however, as with VAT and SCAT, the variability seems to be increased when there is a more unfavorable outcome. Bradshaw and Caan [18, 19] saw increased mortality in patients with sarcopenia and increased SCAT in nonmetastatic breast cancer patients. It remains difficult to compare various cancer entities and different metastatic stages, especially as it is well known that sarcopenia and cachexia are very variable between different cancer entities [4].

In our study, LTW values were increased in patients with a mixed response and those with PD in comparison to patients with a response to treatment. This might be an indicator of anasarca, as CTI is associated with cardiotoxicity and heart failure [43], although ascites, heart failure, and other signs of cardiovascular toxicity were not documented in any of the patients.

A limitation of this study is the small number of patients. Melanoma patients were prospectively recruited and monitored for their responses to the therapeutic interventions, but the MRI examinations were analyzed in retrospect. Thus, patients were not matched for sex, baseline weight or other variables. The exposure to different environmental factors, exercise and food intake, among others, could interact with systemic reactions to CIT. This makes it difficult to correct for different body compositions at baseline, individual food intake, and lifestyle, as well as effects of the immunomodulatory therapy.

## Conclusion

Noninvasive monitoring of the ancillary metabolic effects of tumor progression showed reduced amounts of visceral and subcutaneous adipose tissue in a melanoma mouse model, as well as increased variation in adipose tissue in CIT-treated metastatic melanoma patients with progressive disease or mixed response. Interestingly, LTW significantly increased in metastatic melanoma patients with a mixed response and those with progressive disease, while no change or even a decrease was determined in patients with a response to CIT. Thus, the results of this study emphasize the importance of the monitoring of image-derived metabolic markers of body composition, especially as checkpoint inhibitor therapies become more widely used in clinical practice.

## Abbreviations

BAT: Brown adipose tissue
BMI: Body mass index
CC: Cancer cachexia
CIT: Checkpoint inhibitor therapy
CTLA-4: Cytotoxic T-lymphocyte-associated protein-4
CT: Computed tomography
LDL-C: Low-density lipoprotein cholesterol
LTW: Lean tissue water
MRI: Magnetic resonance imaging
PD-1: Programmed death-1
SCAT: Subcutaneous adipose tissue
TAG: Triacylglycerols
VAT: Visceral adipose tissue
